# Early identification of familial hypercholesterolaemia in general practice using patient-specific reminders: focus group with General Practitioners

**DOI:** 10.1186/1472-6963-14-S2-P97

**Published:** 2014-07-07

**Authors:** Jennifer Tranter, Joe Kai, Nadeem Qureshi

**Affiliations:** 1 Division of Primary Care, University of Nottingham, Nottingham, UK

## Background

Familial Hypercholesterolaemia (FHc) is a common inherited disorder, leading to raised serum cholesterol evident from the first year of life. One in 500 people are affected by the minor heterozygote form of this condition, of which an estimated 85% are unidentified. National English guidelines, produced by NICE, highlight the importance of cholesterol screening and FHc identification in prevention of coronary heart disease [1,2]. However, evidence based approaches to support guideline implementation are under developed. The incorporation of computerised patient specific reminders is thought to have clinical utility in the identification of patients with genetic conditions but as yet not evaluated in primary care for FHc.

## Materials and methods

In order to develop computer based prompts are acceptable to general practitioners a focus group was held for general practitioners from the Inner City Derby area, of which the outcome would inform the design of a future feasibility study to assess the use and impact of such reminders for FHc in clinical practice.

## Results

General practitioners were in favour of specific patient reminders for early identification of individuals at risk of FHc. Emerging themes included brevity, clear and succinct statements, explicit actions that linked specific screening tests to specific diseases and the need to tailor different GP clinical systems.

## Conclusions

Clinicians need to be alert to the prevalence of FHc in clinical practice. Based on results from both the focus group and preliminary work, PSR’s were considered to be an overall effective method of early identification of FHc in general practice and therefore considered relevant in pre-trial work to inform substantive research in the early identification of FHc in primary care.
